# Estimating summary statistics for electronic health record laboratory data for use in high-throughput phenotyping algorithms

**DOI:** 10.1016/j.jbi.2018.01.004

**Published:** 2018-01-31

**Authors:** D.J. Albers, N. Elhadad, J. Claassen, R. Perotte, A. Goldstein, G. Hripcsak

**Affiliations:** a Department of Biomedical Informatics, Columbia University, 622 West 168th Street, New York, NY, USA; b Department of Neurology, Columbia University, 710 West 168th Street, New York, NY 10032, USA; c Value Institute, New York Presbyterian Hospital, 601 West 168th Street New York, NY 10032, USA

**Keywords:** Electronic health record, Kullback-Leibler divergence, Summary statistic, phenotyping, Laboratory tests

## Abstract

We study the question of how to represent or summarize raw laboratory data taken from an electronic health record (EHR) using parametric model selection to reduce or cope with biases induced through clinical care. It has been previously demonstrated that the health care process (Hripcsak and Albers, 2012, 2013), as defined by measurement context (Hripcsak and Albers, 2013; Albers et al., 2012) and measurement patterns (Albers and Hripcsak, 2010, 2012), can influence how EHR data are distributed statistically (Kohane and Weber, 2013; Pivovarov et al., 2014). We construct an algorithm, PopKLD, which is based on information criterion model selection (Burnham and Anderson, 2002; Claeskens and Hjort, 2008), is intended to reduce and cope with health care process biases and to produce an intuitively understandable continuous summary. The PopKLD algorithm can be automated and is designed to be applicable in high-throughput settings; for example, the output of the PopKLD algorithm can be used as input for phenotyping algorithms. Moreover, we develop the PopKLD-CAT algorithm that transforms the continuous PopKLD summary into a categorical summary useful for applications that require categorical data such as topic modeling. We evaluate our methodology in two ways. *First*, we apply the method to laboratory data collected in two different health care contexts, primary versus intensive care. We show that the PopKLD preserves known physiologic features in the data that are lost when summarizing the data using more common laboratory data summaries such as mean and standard deviation. *Second*, for three disease-laboratory measurement pairs, we perform a phenotyping task: we use the PopKLD and PopKLD-CAT algorithms to define high and low values of the laboratory variable that are used for defining a disease state. We then compare the relationship between the PopKLD-CAT summary disease predictions and the same predictions using empirically estimated mean and standard deviation to a gold standard generated by clinical review of patient records. We find that the PopKLD laboratory data summary is substantially better at predicting disease state. The PopKLD or PopKLD-CAT algorithms are not meant to be used as phenotyping algorithms, but we use the phenotyping task to show what information can be gained when using a more informative laboratory data summary. In the process of evaluation our method we show that the different clinical contexts and laboratory measurements necessitate different statistical summaries. Similarly, leveraging the principle of maximum entropy we argue that while some laboratory data only have sufficient information to estimate a mean and standard deviation, other laboratory data captured in an EHR contain substantially more information than can be captured in higher-parameter models.

## 1. Introduction

Electronic health record (EHR) data offer us the opportunity to carry out clinical research on a broad population relatively quickly while minimizing both the financial and human costs because the data are collected for health care. However, because these data are collected for health care and not research they actually represent our observation and actions on the patient rather than the patient him- or herself. Data tend to be collected when patients are ill, for example. We therefore must transform the raw EHR data to a form that is useful for clinical research. One approach is called phenotyping [10,1], which maps the raw data to intermediate states like inferred clinical conditions that are then used in research. Phenotyping may be done manually as a set of rules or queries that assert a state based on raw data [10–14], or it may be automated using machine learning [15–19]. Continuous values like creatinine levels and glucose levels are measured longitudinally, usually at irregular, sparse intervals with a very wide variation among patients in number and spacing of measurements. Providing input to phenotyping algorithms is a challenge because each of the many laboratory and other continuous measurements can be seen as multidimensional (one dimension for each feature) with the number and timing varying among patients. Moreover, many machine learning techniques such as topic modeling only accept ordinal or categorical variables as input, usually focusing on note content and the presence of laboratory measurements. Laboratory data, are important to include in phenotyping because they contain relatively objective information. And while the mere presence of a test has a good deal of information, the addition of a quantification of the magnitude of the test is also important because the magnitude of many laboratory tests are the diagnostics used to define many diseases. A number of simple summarization techniques have been employed, such as using the presence, last value, the median, the mean, the standard deviation, or similar variations. These summaries assume that the important information in the measurements can be conveyed in one or two parameters (e.g., mean and standard deviation). The best summary may depend upon the variable, yet it is unclear how the summaries used in phenotyping are currently selected or what should be selected. For high-throughput phenotyping the selection of a summary technique would have to be automated given the number of potential variables and phenotypes.

Our ultimate goal is to develop an algorithm that can summarize the raw, continuous, inherently noisy, outlier-ridden, biased EHR data such that it emerges as a low-dimension summary that is free of biases, outliers, and other complexities, ready to be used by current machine learning techniques. Moreover, because the point is to help advance high-throughput phenotyping, we also address the problem of scalability. For example, when a problem related to a specific continuous variable is studied, the data from normal and diseased individuals can be studied, thresholds can be extracted from clinical guidelines, and physiologic understanding can used to devise a summary of the laboratory variable. When thousands of variables or diseases are studied at once, then a more automated approach is necessary. The problem is especially challenging when we consider that the variables may be non-Gaussian, that there may be subpopulations beyond the two primary ones—normal and diseased—and that groups of patients may be measured in different clinical contexts.

Our motivation for devising a method for automatically summarizing laboratory data to be used in computational tasks such as phenotyping evolved from four directions: (i) our work on health care process and phenotyping where we observed and documented how the health care influences, confounds, and highlights features that are observable from EHR data [4,1,20,2,21,5,22]; (ii) our Bayesian approach to estimating personalized, time dependent hazard functions that predict the onset of chronic kidney disease—the functions used to model and represent the data were chosen to be Weibull rather than the more standard Gaussian distributions because of the properties of EHR data [18]; (iii) our intuition that the processes generating health care data are relatively sparse [23] and may be summarized and modeled by large contributions from a few dominant features rather than a small contributions from all possible features; and (iv) our work translating phenotypic information to clinical settings where it became clear to us that more simple representations of data, e.g., via single, parameterized families, are more understandable and hence more useful for clinicians than black box prediction [24,25]. In essence, we wanted to find a way to minimize *garbage in* for machine learning methods, to translate laboratory data to a summary that was simple, faithful, interpretable all while minimizing the amount of human effort necessary to clean and summarize the data and therefore minimizing the resources needed to use EHR data in a high throughput setting.

While we followed the above path to this paper we are certainly not the first or only people using complex medical data, or complex data generally [26–29]; there are many other data preprocessing approaches and issues that we don't address here that are important to discuss, including data transformations, preprocessing using clinical knowledge or practice, temporal information, and the use of raw EHR data for phenotyping. Transforming data to a more convenient coordinate system or distribution is one common method used to make complex data easier to handle and more likely to produce more robust results. The Box-Cox transformation [30], which is a power transform [31], is an early method for transforming non-normal data to more normal data so that statistical analysis such as linear correlation can be done more reliably and with less bias. Similarly, general linear models [32,33] depend on transforming the response variables into a space that allows for a linear model to be estimated from diverse predictor variables. In the biomedical domain some researchers have devised more complex transformations of complex medical data to concepts such as anchors [15,16] that are likely to generalize across institutions. While it is common for authors to detrend the data in relatively standard ways [34,35,21], clinical knowledge is sometimes used to preprocess data in a relatively automated way. For example, some have used clinical patterns to discover nominal values [6], while others have worked to devise methods for finding normal ranges of laboratory data [36] and used that information to transform the data into a more practically useful format [37]. Similarly, clinical insight is sometimes used to adjust and transform ICU data in a laboratory-measurement-specific manner [38]. Sometimes data preprocessing is done in a particularly disease-specific way, e.g., [39,40]. Another approach is to standardize data format and quality, e.g., OHDSI [12] represents an effort to create world-wide and standardized health care data bases. These efforts address general data quality and standards but may not address health care process biases explicitly. Time is a crucial property of laboratory data. One issue is whether or not to include time at all. Most early EHR studies to not, and its inclusion depends largely on the questions be asked, the systems generating the data, and the data being used. Another issue is how to represent or parameterize time [41,19], a preprocessing choice that can have a significant impact on what results can be found [42]. But because all EHR data have missing values in time, an ever-present issue is how to incorporate time [43], a question often addressed by framing the data through the lens of missingness [44–47] or imputation and interpolation. For example, some authors use missingness of data as a feature [48,49,7] that can be used to define phenotypes. But more often researches focus on imputation schemes, or methods for interpolate missing values [50,51,21,52-54]. And finally, some phenotyping methods just use essentially raw, unaltered EHR data [55,19,56] with the assumption that the models are flexible enough to manage and model the data complexities automatically.

Together these results point to two high-level choices when preparing EHR data for phenotyping or related applications: use pre-processed or raw data; how and whether to use time in the analysis. In this paper we address the first choice. We do come down on the side of using preprocessed data—the method developed in this paper is a time agnostic method for summarizing laboratory data automatically based on EHR data, producing a numeric or categorical summary that can then be used in phenotyping or similar applications. Our method generates a laboratory variable summary that reveals useful information about the variable despite clinical subpopulations, varying contexts, and bias due to the health care process.

## 2. Methods and materials

### 2.1. Data sources

The study was carried out using two cohorts from different contexts. The first includes EHR data collected during a stay in a neurological intensive care unit (ICU) from patients who are comatose and tube-fed. The second cohort (AIM) comprises the entire longitudinal record of patients who visit regularly the Ambulatory Internal Medicine outpatient clinic, and includes all outpatient visits, hospital visits, ICU stays, emergency department visits, etc. The health care processes underlying these data are very different: in the ICU cohort the glucose data are sampled at approximately regular intervals with noise largely independent of the overall health state of patients, while in the AIM cohort the data are sampled primarily during visits, distributed through time as patients gets sick, or as part of screening and chronic disease monitoring.

#### AIM

We extracted approximately 14,000 patient records from the NewYork-Presbyterian Hospital (NYPH) clinical data warehouse restricted to patients that have visited the NYPH Ambulatory Internal Medicine clinic at least 3 times between September 1990 and September 2010. The full longitudinal records including all inpatient, including ICU, and outpatient data for these patients were gathered. From these records we collected a set of 64 frequently ordered laboratory tests.

#### ICU

We selected 814 patients who were in the neurological ICU, were comatose, tube fed, and had at least 25 measurements. We restricted this data set to include only the time spent in the ICU. In this setting we only consider glucose measurements. In the ICU glucose measurements are generally collected between four and six times a day so 24 measurements represents between four and six days.

### 2.2. Information criterion-based model selection

Our algorithm is based on information criterion model selection method [57,8,9] relying on the Kullback-Leibler divergence (KLD) [58]:

(1) $$\text{KL}(p,q)=\int p\log\frac{p}{q}d\mu$$

where *p* and *q* are probability densities and *μ* is the Lebesgue measure. Intuitively, the KL-divergence between *p* and *q* is interpreted as the information lost when *p* is approximated by *q*. The KL-divergence is used in many formulations of model selection, including Akaike information criterion and Watanabe-Akaike information criterion [57,8,9].

The PopKLD algorithm begins with a non-parametric probability distribution estimate, the kernel density estimate (KDE), *p*, of the population laboratory data, *U_p_*. The KDE, being non-parametric, has hundreds of parameters with no clear interpretation and therefore represents the data very well without summarizing the data any more compactly than the data themselves. Next we approximate *p* with a parameterized probability distribution, for example, a log-normal distribution, creating the approximation distribution, *q*, with two or three meaningful parameters. Finally, we use the KL-divergence to quantify what information is lost when we approximate the non-parametric distribution *p* with the parametric summary distribution *q*. *Summarizing*, the PopKLD algorithm uses the KL-divergence to select the parametric models that minimize the loss of information lost when approximating the non-parametric model *p* with the parametric model *q*. Similar methods are used to derive Akaike information criterion and other IC techniques [8]. The list of parametrized models we use in this paper are described in the Appendix.

### 2.3. Population to individual KL-divergence model selection methodology

**Fig. 1:**
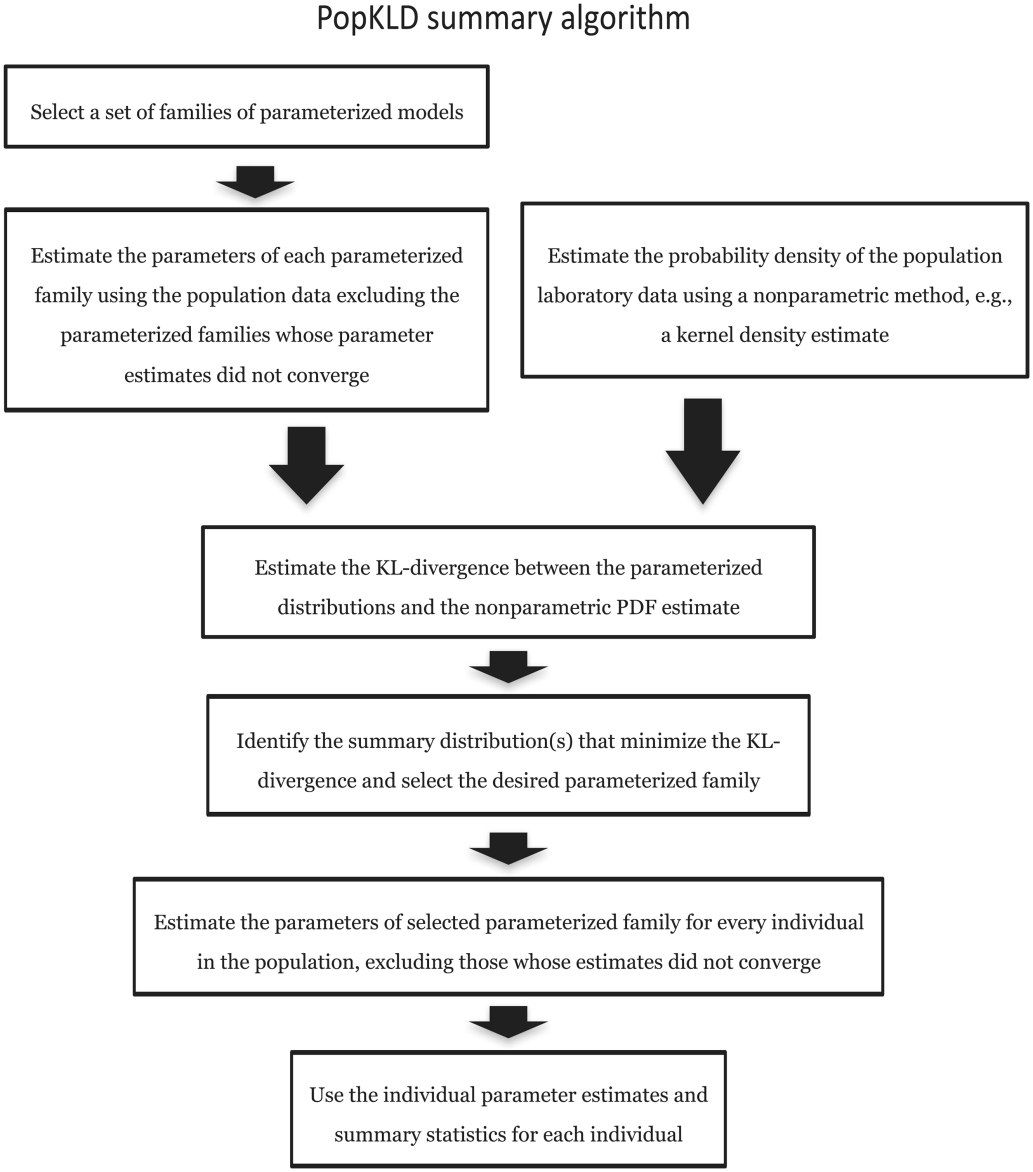
A graphical picture of the PopKLD algorithm for creating a statistical summary of patient laboratory data.

The primary goal of this paper is to construct an *automated, generalizable* algorithm for computing a patient laboratory data summary that is insightful and interpretable, minimizes the information lost by parameterizing the data while minimizing or accounting for bias due to the health care process [1,2,4–6,59]. Our algorithm, the PopKLD algorithm, algorithm has seven steps and is shown in Fig. 1. *First*, select a set of families of parametrized models. *Second*, estimate the parameters of each model using the population data. *Third*, estimate the probability density function (PDF) of the population data non-parametrically using a kernel density estimate (KDE). *Fourth*, estimate the KL-divergence between each parameterized model of the population laboratory data and the KDE of the population laboratory data. *Fifth*, identify the families of distributions that minimize the KL-divergence and select a parameterized family for creating the summary. *Sixth* estimate the model parameters for every individual in the population, taking care to exclude individuals whose parameter estimates do not converge. And *seventh*, use the model parameters as patient summaries for the given laboratory variable.

#### Algorithmic output

The output of the PopKLD algorithm includes three collections of estimates. The first collection includes the parameter estimates for a set of 11 parameterized families, e.g., a GEV, a Gaussian, etc., listed in the appendix, for a population of laboratory values. The second collection includes the KL divergence between the parameterized estimate of population of laboratory data and Kernel Density Estimate of the same data. We use these estimates to select the summary distribution. And the third collection is the summary: the parameter estimates of the selected distribution, e.g., location, shape and scale if the selected model is the GEV, for every individual in the population. These parameters act as a summary for the patient using the model that best resembles the population.

The output of the PopKLD-CAT is a discretization of the output of the PopKLD algorithm. For example, consider the situation where the PopKLD algorithm selected the Gaussian as the most representative parametric model and the PopKLD-CAT discretization specified two categories, high and low, or above and below the 50th percentile. In this case the PopKLD algorithm would generate an estimate of the mean and variance for every individual and the PopKLD-CAT algorithm would discretize the 2-tuple of mean and variance from a continuous value to one of four pairs of categories indicating whether the mean and variance were above or below the 50th percentile of the distribution of mean and variance.

### 2.4. Algorithmic assumptions and limitations

At a high level, EHR data come into existence, or are generated, by two noisy, nonstationary processes, (i) physiology or health, including pathophysiology, and (ii) the health care process that intervenes and *measures* the individual. Usually we do not know very much about the state of these processes, e.g., we do not have measurements that can determine, to a high degree of accuracy, detailed physiology, and we not have a good way of characterizing how people are measured, e.g., ICU measurements are a mix of clinical need and clinical protocols. Moreover, for a given person these processes can change, e.g., measurement and intervention happen very differently in an ICU than in an outpatient setting.

Nevertheless, when we use these data we generally consider these processes to be represented by a model; in the most simple case we represent EHR lab data by simple parametric model, e.g., Glucose measurements represented by a Gaussian. But we know that the chosen model is not measured the same all the time, and we know that the model parameters must change because health changes. Furthermore, we know that as health changes, and model parameters, e.g., mean and variance, change, measurement can change as well—people get sick, are measured frequently, then they get better and are measured infrequently. Even further, we know the population can be diverse, e.g., the EHR could capture a person only in a healthy state such as a person whose measurements begin in their 20s and end in their 40s without a serious injury or change in health state. What this all means in the *simplest* case is that for *single variables*, e.g., glucose, in the EHR include a mixing of unknown models, e.g., distributions, that change in ways only sparsely measured according to measurement processes that themselves are represented by a similar mix of complex and simple processes, e.g., inpatient versus outpatient measurement patterns or measurement patterns driven by different health states. When modeling these data, it is useful to consider the processes that generate these data, and the assumptions we make when we model those data. Relative to the simple situation where we focus on a single laboratory measurement type we assume that there are four broad mixing scenarios for EHR data:

1. The EHR is a mixture: every individual'ss data are generated by wildly different, but distinct individual distributions; e.g., every individual can be represented by a single, unique, distinct distribution, e.g., a Gaussian with a particular set of parameters, but no individual is the same.
2. The EHR is a mixture: every individual?s data are generated by different mixture different distributions; e.g., a given person can be represented by a distinct and unique a mixture of distributions, but no individual is the same mixture.
3. The EHR is not a mixture: every individual?s data are generated by roughly the same distinct individual distribution; e.g., every individual can be represented by one distribution and with relatively similar parameters.
4. The EHR is not a mixture: every individual?s data are generated by the same mixed distribution; e.g., every individual can be represented by the same mixture of distributions with roughly similar parameters.

EHR data can potentially be at least any of these above cases and any transition or mixing of these cases. We don't know the nature of the mixing or how complex the generating function is given today's measurement capabilities.

The reason why the mixing of distributions is important here is that mixing distributions can both create the same distribution or it can create a distribution of a different or larger class of distributions; e.g., mixing exponential distributions with random parameters can result in both a different exponential distribution or more likely a distribution that is a super-class of exponential distributions, a gamma distribution. Implying that a the best model for a population is not necessarily the best model for the individuals making up that population. Moreover, that mixing distributions or generating processes does not necessarily retain the same distribution or generating process gives us leverage to understand the generating processes and formulate hypotheses for productive ways to model and use EHR data. For example, we know that when cases (1) and (2) are extremely diverse and varied, it is likely that the population model and the individual models will not be the same. Cases (3) and (4), the population models will be the same as the individual models, but the population and individuals will not be well represented by a single parameterized model for case (4). The grey areas are the transition between cases; e.g., the transition between cases (1) and (2) where the population is not very diverse to cases (3) and (4) where the population is somewhat diverse may be modeled by models for either scenario. Our hypothesis or assumption, is that EHR data are often close enough to case (3) that our algorithm will work for summarizing laboratory data—that we can represent a given laboratory variable with a single distribution, and that while there is variation within the population due to many factors, there is not so much variation that the population model that it isn't also a *relatively good model of the individual*. While it is likely that partitioning the data by the contexts of collection will make our hypothesis more true, and while we can construct counterexamples to our intuition, here we are interested in the high-throughput case where we can automate an algorithm that is *usually* sensible. It is not possible to test our assumptions explicitly because we want to leverage the large number of sparsely measured people in an EHR, and most individuals are measured too sparsely to accurately draw distinctions between what distribution best represent them. Instead, we are left evaluating the sensibility of the assumptions by evaluating the effectiveness and face-validity of our method. But it is important to understand the assumptions that underly our algorithm because it will help understand when the algorithm is likely to fail. E.g., it is likely that algorithm will not work well when the population is particularly diverse, but we do not know how much diversity is too much. However, given the results in this paper, it is likely the algorithm can handled substantial diversity.

### 2.5. Transforming PopKLD summaries into categorical variables

**Fig. 2:**
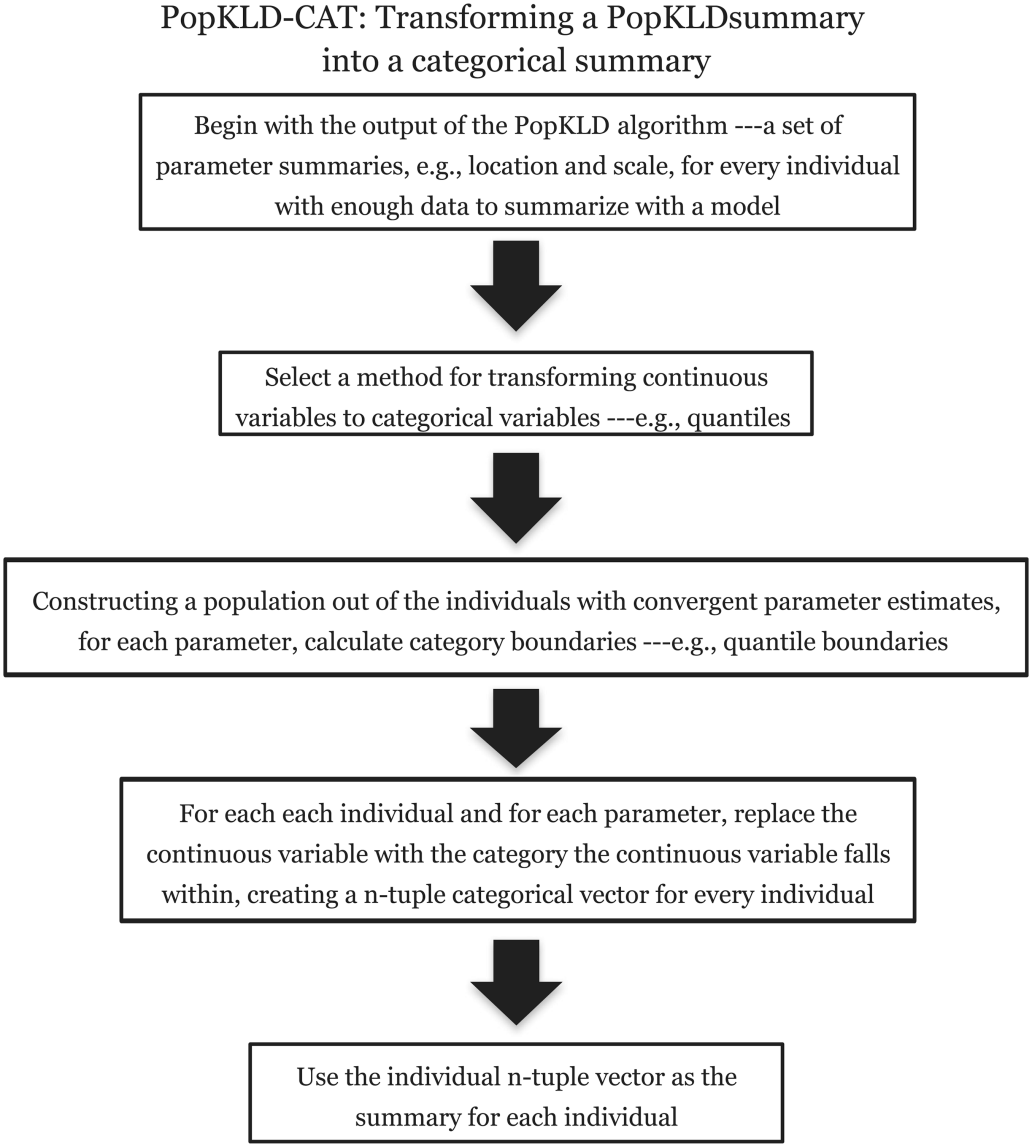
A graphical picture of the PopKLD-CAT algorithm that translates the continuous PopKLD patient laboratory data summaries into categorical variables that can be used in situations where categorical variables are necessary, such as topic modeling.

Some machine learning methods that are used to automate the task of defining phenotypes or cohorts require discrete or categorical variables. Therefore, to be useful in this circumstance the PopKLD algorithm must allow for a mapping to categorical variables. We translate the continuous PopKLD summary into an *ordinal* summary using the PopKLD-CAT algorithm, shown in Fig. 2, in four steps. *First*, choose a method for translating continuous model parameters into ordinal categories such as deciles [60]. *Second*, given the distribution of parameter estimates, calculate the category boundaries. *Third*, for individuals whose parameter estimates converged, map each parameter to the category, here decile, it falls under; e.g., if the mean is in the 1st decile and the standard deviation is the 8th decile, then the individual would be represented by the 2-tuple vector (1,8). And *fourth*, use the new categorical representation of the patient for the chosen task such as topic modeling.

### 2.6. Maximum entropy for evaluation

The concept of entropy maximization in the context of machine learning is generally used for selecting a probability distribution that best represents data according to the principle of maximum entropy. The principle of maximum entropy states, subject to data and various technical assumptions, that the distribution that maximizes entropy is the distribution that represents the current system most accurately with the fewest assumptions. In this way, the maximum entropy is just another property, like maximizing log-likelihood, minimizing mean square error or KL-divergence, etc., that can be used to select a model or estimate optimal parameters. But, the entropy maximization has an intuitive interpretations that most other metrics do not have and we will leverage two of these interpretations our evaluation. *First*, the maximum entropy model is the model that minimizes the use of assumptions used to estimate the model parameters. Intuitively this means the maximum entropy model is the least overfit model we could use while maximizing the information we do have about the system. *Second*, many natural systems are observed to maximize entropy, implying that nature has found the best way to select a probability distribution is using the maximum entropy principle. We use these ideas in the following way: if the PopKLD algorithm, which is not using the principle of maximum entropy, either selects or rejects the maximum entropy model choice, this helps us interpret the meaning of the PopKLD selection process and the process generating the data.

The maximum entropy distribution for a data with one constraint, a mean, is the uniform distribution. The maximum entropy distribution with two constraints, a mean and a standard deviation, is a Gaussian distribution. The maximum entropy distribution with three constraints, mean, standard deviation, and a linear relationship between mean and standard deviation [61] is a Gamma distribution. For most of our laboratory data, assuming we know nothing about the data but are able to estimate a mean and a standard deviation from data, *maximum entropy* predicts that a Gaussian distribution will be the best distribution to use for representing the data. *There is one special exception* to the prediction that a Gaussian distribution will be the most useful: we know from physiology that glucose dynamics have a linear dependence between mean and standard deviation [62,63], imply that for glucose, maximum entropy predicts that the Gamma distribution will be the best distribution to use for summarizing glucose.

We use the predictions from entropy maximization as a guidepost for understanding the meaning of the PopKLD model selection by comparing adherence or deviation from the maximum entropy prediction. For example, if the PopKLD algorithm selects a distribution that has a parameter for the tails of a distribution, e.g., the generalized extreme value distribution (GEV) [64], then that implies that the data include more than just information about the mean and standard deviation, but information about higher order features of the distribution such as information about the tail of the distribution. In contrast, if the PopKLD algorithm selects a Gaussian distribution, it is likely that the data mostly contain information limited to estimating two parameters, mean and standard deviation.

### 2.7. Evaluation of the population KL-divergence model selection method

We evaluate the PopKLD algorithm in two ways, by testing that the PopKLD selected models preserve physiologic relationships we know a priori are present and by performing a phenotyping task were we use the PopKLD-CAT algorithm to identify patients with diabetes using glucose, patients with chronic kidney disease using creatinine, and patients with pancreatitis using lipase all compared against a gold standard created by an physician review of patient records. *It is important to note that while we use a phenotyping task to evaluate the PopKLD algorithm, the PopKLD algorithm is not meant to be used as a phenotyping algorithm*. We use the phenotyping task as an evaluation because it directly reveals how the PopKLD algorithm works and is a rigorous evaluation because it requires the PopKLD algorithm to perform without the aid of other information normally used in machine-learning-based phenotyping algorithms.

#### 2.7.1. Evaluation one PopKLD summary's preservation of known physiology and comparison with principle of maximum entropy predictions for intravascular glucose

The first evaluation of the PopKLD model selection algorithm is a demonstration that the PopKLD algorithm preserves known physiologic relationships that are hidden using more common summaries of laboratory data such as mean and standard deviation. Physiologic relationships within a single laboratory variable are not always easy to come by so this evaluation technique cannot be applied to all laboratory variables, but when it can be, it is powerful. We further reinforce this evaluation by applying the PopKLD algorithm in multiple contexts—here we apply the PopKLD in two contexts, the EHR and the ICU for the same laboratory variable. By doing this we achieve two goals. First, we can observe how measurement context, how mixing measurement contexts, or potentially how the health care process, may impact the laboratory measurements collected. Second, we can observe and quantify how the PopKLD algorithm copes with and adapts to biases such as mixing measurement contexts or the health care process—validating the robustness of the PopKLD algorithm relative to changes in data collection context.

The physiologic relationship we leverage here is related to glucose: when carefully measured, the mean glucose should be linearly related to the standard deviation of glucose [65,66,3,62]. In this situation, because of the linear relationship between mean and standard deviation, we have an additional *independent* model selection that will help buttress our results, maximum entropy. Given the linear relationship between mean and standard deviation, maximum entropy *predicts* that best parameterized model will be the *gamma* distribution [61]. Armed with this prediction we gain two extra insights. First, if the PopKLD selects the same model that maximum entropy predicts, the consistency is reassuring and suggests that PopKLD is selecting a meaningful model to generate a summary. Second, if PopKLD does not select the same model that maximum entropy predicts, this may implicitly imply that the measurement function is dependent both on physiology and the health care process. Nevertheless, showing an explicit dependence on the health care process requires more work as the deviation from maximum entropy distribution may also be due to other factors.

#### 2.7.2. Evaluation two: using PopKLD and PopKLD-CAT for a phenotyping task

The first evaluation is a deep analysis into the modeling of a single laboratory variable, but it does not address generalizability, application of the method to categorical or ordinal variables, or demonstrate practical usefulness or clinical tasks such as phenotyping or cohort selection. The second evaluation is designed to address these limitations by applying the PopKLD algorithm to more than 62 laboratory variables, applying the PopKLD-CAT algorithm to the results to generate categories, and then evaluating the usefulness of the categorical summaries on three different laboratory variables.

The second evaluation focuses on evaluating the PopKLD-CAT algorithm in the context of identifying phenotypes and for this we restricted the evaluation to AIM data. We begin with three disease and laboratory data pairs, diabetes and glucose, chronic kidney disease and creatinine, and pancreatitis and lipase. In a real phenotyping setting, we would use many more variables and data; this evaluation is not about phenotyping but about evaluating what information can be gained in a phenotyping task by using the PopKLD laboratory summaries compared with mean and variance of the same laboratory variable. We then evaluate how successful the PopKLD selected model is at summarizing an individual patient's raw laboratory data by using the PopKLD summary to identify patients with a given disease for diseases that are defined by laboratory values that are elevated. This evaluation is carried out in five steps.

##### Step one

We apply the PopKLD algorithm using three models to summarize the data, generalized extreme value (GEV), log-normal, and empirical mean and standard deviation. We selected the GEV and the log-normal distributions because for glucose, lipase, and creatinine, the PopKLD algorithm selects the GEV as the best model distribution, followed by the log-normal distribution. The empirical mean and standard deviation are used in comparison because they are often used to summarize laboratory values from phenotype studies, cohort selection, etc. So, the comparison is between the summary provided by the PopKLD algorithm and the state of the art.

##### Step one outcome

The outcome of step one is a set of summary parameters for three models, GEV, log-normal, and empirical mean and standard deviation for every AIM individual whose model estimate converged in maximum likelihood, organized by decile.

##### Step two

We ordered patients according to parameter deciles and then collected two groups of patients per model (three for the GEV) according to having all parameters in either the 1st or 10th deciles. For the GEV we split each of those sets of patients into two more categories according to whether the shape parameter was positive or negative; the shape parameter controls the direction of the tail of the distribution.

##### Step two outcome

The outcome of step two is a collection of sets of patients who had high and low values of the laboratory summary variables.

##### Step three

Beginning with the groups of patients collected in step two, we selected 15 random patients from each subgroup for manual gold standard curation by the clinician. For example, we selected 15 patients whose mean and standard deviation were both in the 10th deciles.

##### Step three outcome

The outcome of step three is a subset of patients for manual review and subsequent creation of a gold standard to evaluate the PopKLD methodology.

##### Step four

We gave a clinician the 30 patients for each model category randomly ordered and blinded and had the clinician manually review the patient's record and identify whether the patients had or did not have one of the given diseases.

##### Step four outcome

The outcome of step four was the creation of the gold standard used to evaluate the PopKLD algorithm.

##### Step five

We estimated the cluster purity [67] of each 15-patient group to evaluate how pure each of the 15 patient groups were relative a given disease. For example, for the log-normal distribution for glucose, the 10th decile group had 15 out of 15 patients with diabetes, achieving a purity of 1; cf. Table 2 for the results.

##### Step five outcome

The outcome of step five is a quantitative evaluation of the PopKLD algorithm against a gold standard for a broad EHR population.

## 3. Results

### 3.1. Summary models for laboratory data types identified by the PopKLD algorithm

The results from the PopKLD algorithm for 64 common laboratory values are found in Table 1; the laboratory values included are split into clinically relevant groupings, including metabolic, blood gasses, whole blood, differential, hepatobiliary, lipids, anemia, cardiac, hormone, inflammatory, vitamin and urinary laboratory values. Recall that all of the laboratory values were collected in the AIM clinic with the exception of one, the ICU glucose. The ICU-restricted glucose is included in an attempt to isolate the data generated primarily due to physiology and with relatively minimal health care process bias due to collection context.

Within Table 1 we would like to focus on five observations. *First*, there is no obvious general rule of thumb for picking a best or most representative distribution for all laboratory data types. All parametric models have laboratory variables that they represent particularly poorly as characterized by a comparatively large KL-divergence while still being among the best to represent other laboratory variables. *Second*, there is diversity in how many models can reasonably model given laboratory data. Some laboratory types have a clear winner among models, e.g., AST and ALT are best approximated by the GEV because the KL-divergence is smaller for the GEV compared to all other models an order of magnitude or more, while others laboratory variables have many models that can represent them well, e.g., urinary pH and T4 have several models whose KL-divergence agrees out to 2 orders of magnitude or more. *Third*, most but not all laboratory measurements deviate from the normal distribution in a substantial way. Only a few laboratory measurements are well represented by the normal distribution. Because of this, assuming normality with laboratory values is generally not a good idea. Moreover, because of the deviation from the maximum entropy prediction, most laboratory variable data have more information than is contained in the mean and variance alone. *Fourth*, often when the laboratory measurements are well modeled by a normal distribution they are also well modeled by several other parameterized models. This may imply that these laboratory measurements have among the least structure or constraints imposed by their generating process—this interpretation is again motivated by ideas from maximum entropy. And *fifth*, one interpretation of model selection is that the selected model is the model most similar to the generating process of the data. In other words, if the PopKLD algorithm selects the GEV, the interpretation would be that the process generating the data is some kind of extreme value process such as measurement restricted to acute illness. While there may be something to this interpretation, we must be careful about drawing too strong a conclusion from this result for two reasons. First, the real generating process may not be well represented by any of the 11 models even by approximation. And second, without a explicit mechanistic reasons and understanding that predicts a particular model selection, we must use care in extrapolating implications of a given model being selected as the most representative. This is important because it relates to our first evaluation methodology and why we buttress our first evaluation of the PopKLD algorithm with other evaluation techniques.

### 3.2. Evaluation one: PopKLD summary's preservation of known physiology and comparison with principle of maximum entropy predictions for intravascular glucose

When EHR data are not influenced by collection context or other health care processes, they should represent the physiology of the patient. In this setting, the PopKLD should select the distributions that preserve physiologic features. To test this we evaluate the PopKLD algorithm in two data collection contexts. First we apply PopKLD to glucose data collected in the ICU, a single context data source. We hypothesize that the ICU data represent mostly physiology because the measurements such as glucose in an ICU are collected largely independent of the state of the patient compared with other EHR data collection contexts. Second, apply PopKLD to glucose from the EHR limited to patients who visit the Ambulatory Internal Medicine clinic, or the AIM clinic. These data represent a mixed context data source because these data include all data for AIM patients, including ICU data, but primarily contain outpatient data. We hypothesize that the AIM data represent a mix of physiology and HCP. In both contexts we show that the PopKLD produces laboratory summaries that preserve know physiology. Specifically, that for glucose, mean and standard deviation are linearly related.

In the case of the ICU data, we can further evaluate the PopKLD algorithm because we can make a prediction. The maximum entropy distribution for *any system* with the constraint that mean and standard deviation are linearly related is the gamma distribution. Therefore, in the context of the ICU, if the ICU data are primarily representative of physiology, we predict that the PopKLD algorithm will select the gamma distribution to best summarize glucose.

#### 3.2.1. PopKLD of glucose collected in a single context

**Fig. 3:**
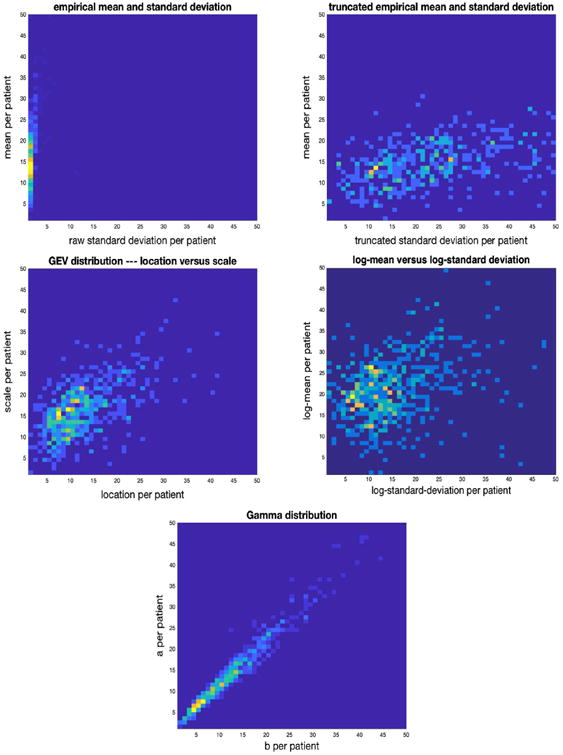
Joint distributions for: mean vs raw standard deviation (top left), mean vs truncated standard deviation (top right), location vs scale—the mean-like and variance-like parameters of the GEV—(middle left), log-normal mean vs standard deviation (middle right), and “a” vs “b” of the gamma distribution for the ICU population (bottom). PopKLD selected the log-normal and gamma distributions as the best models and both reproduce known physiology well.

**Fig. 4:**
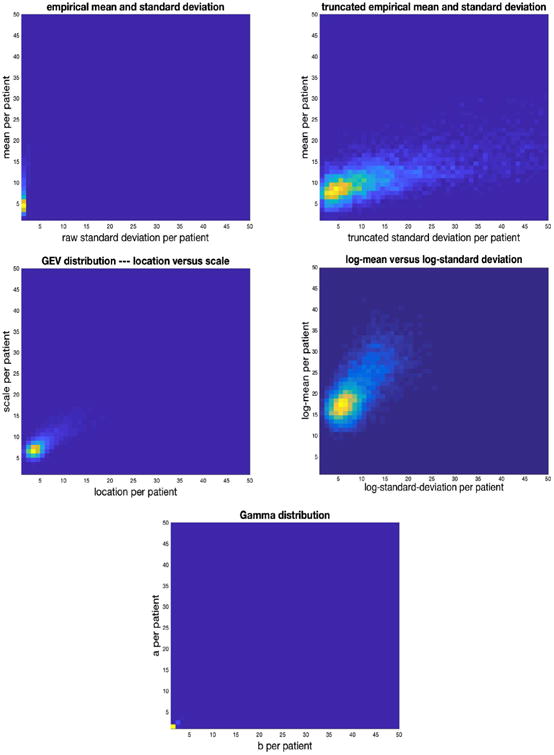
Joint distributions for: mean vs raw standard-deviation (top left), mean vs *by hand* truncated variance (top right), location vs scale—the mean-like and variance-like parameters of the GEV—(middle left), log-normal mean vs standard deviation (middle right), and “a” vs “b” of the gamma distribution for the AIM population (bottom).

PopKLD selects the lognormal and the gamma distributions as the best summaries for glucose. In both cases the known physiologic relationship was revealed and both PopKLD and the independent maximum entropy predictions agree. Fig. 3 shows the relationship between the empirical mean and standard deviation, both raw and truncated by hand, meaning we removed all cases where the standard deviation was greater than 2000, the mean-like and standard-deviation-like quantities for the log-normal distribution, the gamma distribution and the GEV distribution. We include the GEV because it is the PopKLD selected distribution for the broad EHR data that we will discuss in the following section and we wanted to show the contrast. The empirical mean and standard deviation reveal no relationship in their raw forms; when we remove the outliers of the mean and standard deviation *by hand*, the physiologic relationship we seek appears (cf “truncated standard deviation in Figs. 3 and 4). This by-hand treatment is not useful in a high-throughput setting and shows how the mean and standard deviation can fail to cope with the health care process. Moreover, this figure is allows for direct observation of the effects outliers have on the robustness of mean and standard deviation estimates. The log-normal and GEV models *automatically* reveal the physiologic relationship we know is present. The gamma parameters, the model predicted by maximum entropy to be the most representative model, reproduce the strongest, cleanest physiologic relationship. The other model the PopKLD selected also reproduced the physiologic signal we know to be present, as did the GEV. This implies that a good-enough PopKLD score may be enough to justify using a given model to summarize data.

#### 3.2.2. PopKLD summary of glucose collected in a mixed context

In the mixed context setting PopKLD selects the GEV distribution to summarize glucose because it minimizes the KL-divergence, but the lognormal remains a plausible summary distribution because it is not far from the minimum KL-divergence. In contrast to the ICU data setting, the gamma distribution—the distribution that we would expect to be selected assuming only physiology—is not among the models selected by the PopKLD to summarize glucose. Fig. 4 shows the relationship between the empirical mean and standard deviation, both raw and truncated by hand where we again removed all cases where the standard deviation was greater than 2000, the mean-like and standard-deviation-like quantities for the GEV, the log-normal distribution, and the gamma distribution. The empirical mean and standard deviation again reveals no relationship in their raw forms; when we truncate by removing the outliers of the mean and standard deviation, the physiologic relationship we seek appears in a much more pronounced way compared to the ICU data. Again, this by-hand treatment is not useful in a high-throughput setting. The GEV and log-normal models *automatically* reveal the physiologic relationship we know is present. In contrast, and as predicted, the gamma parameters only very weakly reproduce the physiologic relationship—the gamma is not a good model for summarizing glucose using mixed context EHR data.

In the AIM data context, the PopKLD-selected the model is not the model we would have picked based on knowledge of glucose physiology, the gamma distribution, but it is the one that preserves physiologic relationship between mean and standard deviation of glucose the most clearly. Moreover, PopKLD, while preserving the physiology, did not select the maximum entropy model. This deviation may be because the generating process is no longer governed by glucose physiology in a dominant way—that the HCP and measurement noise may be contributing in nontrivial ways to the data in addition to the biology we observe. The empirical mean and standard deviation are unstable, obscure the physiology, and are largely useless as was the case with the single context ICU data. We hypothesize that the reason the log-normal and GEV worked better than the gamma is that the GEV and log-normal distributions are handle outliers very well, while the gamma is lost in outlier havoc, but this remains a hypothesis.

### 3.3. Evaluation two: using PopKLD and PopKLD-CAT for a phenotyping task

The results of evaluation two, the phenotyping task, are shown in detail in Table 2. We would like to focus on six results. *First*, PopKLD appears to work well for selecting a parameterized model to summarize laboratory data for use in the task of phenotyping patients or identifying cohorts of patients with a laboratory-definable disease. Again, the PopKLD algorithm is meant as a data preprocessing algorithm instead of an algorithm for phenotyping, but the PopKLD summary of laboratory data does appear to supply the information needed identify different patient phenotypes more accurately than mean and standard deviation. *Second*, the mean and standard deviation work poorly as summary variables and can only reliably help determine absence of a disease. We suspect that mean and standard deviation work poorly because they are not robust statistics; it is possible that applying m-estimators or other robust statistics tools [68] would make mean and standard deviation more useful. *Third* for all of the models it is apparently easy to detect absence of a disease when the presence of the disease is a defined by a high value of the laboratory value. The implication is that outliers in this situation are biased toward being too high; this may not always be the case for every laboratory measurement. *Fourth*, the PopKLD selected model, the GEV generally does well relative to the purity against the gold standard, although it underperforms on the identification of pancreatitis. *Fifth*, the GEV, aside from the mean-like location and the standard deviation-like scale, has a tail-controlling parameter called shape and sometimes the shape parameter matters for helping to identify a disease. For example, in the case of CKD, negative shape, or a left tail, helps better identify patients with CKD. That the GEV has more parameters to leverage can be an advantage—here enforcing a negative shape implies a hard upper bound on laboratory measurements, decreasing the likelihood of high outlier, while being able to retain information about high mean and standard deviation-like parameters. And *sixth*, the second best performing model according to the PopKLD, the log normal, generally performs well, especially in the case of pancreatitis where it outperforms the GEV. It is likely that using more than one model summary, e.g., the top three PopKLD models, may be helpful in high-throughput applications as there may be little cost in calculating such quantities and using them in phenotyping schemes. Constructing a model averaging [9,69]or ensemble learning [70–72] approach here may be very useful. Another less redundant option would include an uncertainty analysis into the PopKLD algorithm; we will cover this option in the discussion.

## 4. Discussion

### Summary

We developed an algorithm, the PopKLD algorithm, for summarizing EHR laboratory data that is automated, generalizable, and robust to context of collection and therefore can potentially be used in high-throughput phenotyping applications. This does not mean that the PopKLD algorithm is a phenotyping algorithm—it is not—but rather that the laboratory summaries estimated by the PopKLD algorithm may be provide more information than mean, standard deviation, or presence/absence, when integrated into a high-throughput phenotyping algorithm. The PopKLD model selection algorithm revealed that context of data collection, e.g., health care process, physiology, etc., potentially may contribute to the data we observe in quantifiable, identifiable ways.

### Modeling electronic health record data generated by physiology and the health care process

We have shown that in different clinical contexts, different parameterized models of data may be appropriate, and we hypothesize but have not proven that the cause is not so much a change in physiology, but a change in the way health is measured in the different contexts. That is, we hypothesize that the health care process causes different but measurable biases in the data and provide more evidence validating this hypothesis. In the ICU, the health care process measurement function does not appear to have a strong influence on glucose measurements. Circumstantially, we observe that we can treat and use ICU data more like continuously sampled physiologic data. In contrast, the process that generates or collects data from outpatient, or mixed outpatient-inpatient settings, does influence the data collected more profoundly. If the reasonably correct model is not chosen, the physiologically expected relationships between parameters are lost. Written differently, it is likely that we must understand something about, or otherwise account for, the generating processes of EHR data in a concrete way if we intend to use EHR data to their full capacity. Moreover, if we ignore the health care process entirely, our results may be highly suspect. The PopKLD algorithm is meant to reduce biases such as the health care process bias, but a deeper understanding of these biases will be necessary to more completely and positively remove them.

### High-throughput phenotyping application

One of the points of the PopKLD algorithm to create a reasonable, interpretable, stable, automatable, ordinal summary of laboratory data that can be used in high-throughput situations where machine learning [17,16,15] is used to categorize humans. Based on the results from the clinical evaluation, we believe that PopKLD will be very useful in phenotyping studies.

### PopKLD not meant to be used by itself

The point of this paper was not to create a laboratory summary algorithm to be used as a single and only summary of an individual. While we did use the PopKLD summary variables as lone summarizations of patients for the clinical evaluation, this is not the intended use. Rather, the PopKLD algorithm was designed with two goals in mind. First, the PopKLD summaries were intended to be used in conjunction with other variables as a stable and accurate summary of a given laboratory value that can be generated automatically for high-throughput applications. Second, the PopKLD algorithm was intended to transform laboratory values from continuous to discrete summaries when necessary for use in high-throughput settings such as topic modeling.

### Striking a balance between accuracy and interpretability

In this paper we developed an algorithm for creating a simple, interpretable summary of laboratory data—PopKLD—and a method for discretizing that summary when necessary—PopKLD-CAT. There are many options for further modifying our algorithms to include more complex methods, including adaptations of Lasso, general linear modeling, Bayesian methods, mixture models, random effects models, etc. These modifications raise the question of the balance between interpretability and accuracy. On one end, if one wants to maximize accuracy of a distributional estimate and doesn't care so much about interpretability or simplicity, then modeling the data with a KDE or a neural network, or a spline, or a mixture model, etc., will provide a more accurate representation of the data almost surely. But often what gained using a more complex model, accuracy of estimation, and is lost is interpretability. For example, mixing only two models together makes interpreting parameters much more complex; e.g., a KDE is, in essence, a mixture of different kernel functions whose parameters are not easily interpretable. Our algorithm development was driven by concrete constraints; we wanted an interpretable, single parametric model representations of the data that was robust, useful on sparsely sampled individuals, and we wanted the selection of such a model to be automatable. The balance we struck has limitations, as discussed in Section 2.4 and we suspect that modifications to the our algorithm that detect, account for, and build out from these limitations will extend interpretability and reduce the level of garbage in within high-throughput phenotyping pipelines.

### Incorporation of uncertainty analysis and model error into the PopKLD algorithm

We did not introduce uncertainty quantification into the PopKLD algorithm because we wanted to simplify the presentation of the core concepts of the algorithm and because we wanted to highlight that it may be useful to use more than a single summary model. Moreover, uncertainty quantification induces many choices that we did not want to highlight or focus on. Nevertheless, it may be useful to preform uncertainty analysis, especially in situations where there are many potentially useful models and only one is desired. Uncertainty analysis is rather simple to incorporate into the PopKLD algorithm in theory, but can become messy in practice. For example, applying the jackknife bootstrap, or bootstrap with replacement on patient, or bag of little bootstraps to the population will allow easy computation of a confidence interval on the KDE of the population [73–75]. The problem is then determining the KL-divergence between two distributions with confidence intervals around them. One solution is to estimate the KL-divergence between the confidence interval bounds that maximize the difference at every point in the support, but there are many other options. Here we took a more simple tactic, we assume the model error is always quite high and do not focus on only the model that minimized the KL-divergence but also consider other models that are near the minimum of KL-divergence. This robustness tactic is well-worn: we treat all the models near the KL-divergence minimum as perturbations of one another in a functional sense, and by evaluating all of them, we evaluate the robustness of PopKLD algorithm relative to selecting any of the models near the KL-divergence minimum. What we observed in our evaluations is that all the models near the KL-divergence minimum did quite well reproducing known physiology and matching the clinician defined gold standard. Because of this robustness to functional perturbations, we are more confident that PopKLD will be useful in a more automatic setting where, for example, the user randomly selects one distribution in the case where there is more than one best distribution.

### Insights from the KL-divergence estimates

We did not include an analysis of all comparisons between the non-parametric KDE models of the population and the parametric models because there are 704 such comparisons. We did look at all the KL divergence estimates and graph combinations we were surprised by a few things: (i) nearly all parametric families approximated some laboratory variables well while almost no parametric families approximated other laboratory variables well; (ii) sometimes a few parametric families fit a laboratory variable very well but differently while the rest of the parametric families fit the laboratory variable miserably; and (iii) sometimes subclasses of distributions provided much better estimates of a laboratory variable, e.g., the Weibull may not resemble the GEV estimate for a given data set, even though the Weibull is a GEV subclass. This later issue reveals the complexity and sensitivity that model estimates can have to the method used to estimate the model parameters. Meaning, different model estimation algorithms with, e.g., different methods for selecting parametric starting points, can arrive at different parametric estimates given the same data. This problem is not new and is a consequence of the difficulties encountered in choosing suitable parameter estimates given that a global optimal parametric estimate that may neither exist nor be easy to find. Because of this it may be useful when describing a model used to pair it with the algorithm used to estimate the model as was done in [76].

### Data requirements of the algorithms

One of the powers of EHR data lies in the size of the population; we increase the data set by increasing the population while every individual remains sparsely measured. For the analysis here we included all individuals with at least five measurements. Generally, most of the individuals in our data set had fewer than 10 measurements per laboratory measurement type but there was variation in the amount of data present per individual across the different laboratory measurements. Our algorithm excludes all individuals for which an parametric model estimate did not converge. This failure to converge was relatively infrequent and was dependent on both the laboratory measurement and the chosen model. Most commonly, the algorithm excluded fewer than 5% of the population, meaning that the algorithm worked well on sparsely measured individuals.

### Deviation from the normal distribution

As a byproduct of our analysis, we observed that the normal distribution is generally, but not always, among the worst representations of laboratory measurements. This has two implications. First, very few laboratory values are well represented by a normal distribution. The likely reason is that the normal distribution has symmetric tails while most physiologic variables have relatively hard lower bounds quite a distance from zero. But, the point is that for many analysis of laboratory values from hypothesis testing to machine learning (e.g. assumed Gaussian priors) include assumptions of normality, and those assumptions are likely quite wrong and may effect the conclusions of those studies. Second, mean and standard deviation may not be very useful quantities to characterize distributions of laboratory measurements. This does not mean that a mean-like centroid quantifying quantity and a standard deviation-like distributional spread quantifying quantity are not useful, just that mean and standard deviation themselves may not always be particularly representative or insightful quantities.

### Empirical estimation and the non-robustness of the mean and standard deviation

It is well understood that the mean and standard deviation are non-robust statistics. These quantities can be made more robust with some effort, but such effort is rarely employed. Instead, we often assume that the non-robustness of the mean and standard deviation will not be so bad as to deeply obscure their meaning. Moreover, we assume that by adding more data we may be able to reduce data quality problems. However, adding more data doesn't help because as more data are added, more outliers are also added at a roughly constant rate. So often the empirical mean becomes similarly or more corrupted as more data are added, not less corrupted. Meaning, our assumption that assuming that the non-robustness of the empirical estimates like a mean may not be so bad, or can be corrected by using more data is not consistent with the data and our understanding of robust statistics. Here we have quantification for how bad this assumption really is—mean and standard deviation failed miserably when used to identify presence of diabetes, chronic kidney disease, or pancreatitis. When using EHR data, it is likely best to either avoid mean and standard deviation when possible or employ robust statistics machinery or to use the method we propose here to select a representation to summarize the centroid and the variance around the centroid.

### Why not use the principle of maximum entropy?

One could ask why not use maximum entropy as the model selection method rather than just as an evaluation method. At this point, it is difficult to estimate the entropy in a meaningful way using standard parameterized families of distributions because it is difficult to estimate the tails of the continuous distributions well in the setting of sparse data, and some of these distributions are sensitive to the tails. But we suspect that if these problems can be addressed maximum entropy would be useful for model selection for models of sparsely measured continuous variables just as it has been shown to be useful in the context of discrete variables [77] and natural language processing [78].

## 5. Summary

We developed the PopKLD and PopKLD-CAT algorithms that admit raw, continuous, inherently noisy, outlier-ridden, biased EHR laboratory data and emerges with a low-dimension summary that is less dominated by health care process biases, outliers, and other complexities, ready to be used by current machine learning technology. The algorithms, meant to be used to preprocess EHR data for use in high-throughput phenotyping and cohort identification algorithms, are easily automated and scalable as the number of laboratory variables and the patient population is increased. The algorithm excludes temporal features of the data, but can produce a robust summary that is either continuous using the PopKLD algorithm or ordinal using the PopKLD-CAT algorithm, pushing the fidelity of laboratory data summaries in such a way to be useful to many machine-learning-based phenotyping algorithms.

## Figures and Tables

**Table 1:** Metabolic laboratory PopKLD model selection estimates were we list the PopKLD selected models. Multiple models are listed if their KL-divergence is a minimum and agrees one two or more orders of magnitude. Note the diversity in which model is selected and how many models are good approximations cross laboratory measurements. The data used were from the AIM clinic data set with the exception of the glucose data collected in the ICU (GLU-ICU).

Summary models selected by the PopKLD for 64 laboratory features					

Basic metabolic		Whole blood		Hepatobiliary	
		
Lab-Context	PopKLD model	Lab	PopKLD model	Lab	PopKLD model
GLU-ICU	Gamma, LogNorm	HGB	Norm, Weibull, Logistic	AST	GEV
GLU	GEV	MCH	Logistic	ALT	GEV
CA	Logistic	MCHC	Logistic	AMY	GEV, LogNorm
CL	Logistic	HCT	Gamma	LIP	GEV
CREAT	GEV	RBC	Norm, Logistic	BLOOD PROTEIN	Weibull, Norm, Logistic
K	LogNorm	RDW	GEV	BILI TOTAL	GEV
MG	Logistic	MCV	Logistic	BILI RECT	Uniform, t
PH	GEV, Logistic, LogNorm	PLT	Logistic	ALB	Weibull, Logistic, Norm
BICARB	Norm	MPV	Norm, Gamma, Logistic, LogNorm, Weibull	ALK PHOS	GEV
BUN	GEV	WBC	GEV, LogNorm, Gamma		
URIC	GEV, Gamma, LogNorm				
CA ION	Logistic, Norm				
HA1C	GEV				
Lipids		Anemia		Cardiac	
		
Lab-Context	PopKLD model	Lab	PopKLD model	Lab	PopKLD model

HDL	GEV, GAMMA, LogNorm	FERRITIN	GEV	CK	GEV
LDL	Gamma, GEV, Norm, Logistic	IRON BINDING CAP	Norm, Weibull	TROPONIN	GEV
TG	LogNorm, GEV	VITAMIN B12	Rayleigh	LACTATE	GEV
CHOL	Gamma	IRON	GEV, Logistic		
Hormone, Inflam, Vitamin, Urine		Differential		Blood Gases	
		
Lab-Context	PopKLD model	Lab	PopKLD model	Lab	PopKLD model

TSH	GEV, LogNorm,	BASOS %	Uniform	BASE EXCESS ART	Rayleigh, LogNorm
T4 FREE	GEV, Gamma, Logistic, LogNorm	MO %	GEV, Logistic	PO2 VEN	GEV, LogNorm
T4	GEV, Gamma, Logistic, LogNorm	LYMPH	GEV, Gamma	PO2 ART	GEV
CRP HIGH SEN	GEV	NRBC abs	t	PCO2 VEN	Logistic, Gamma
ESR	Logistic, GEV, Gamma	NRCB %	Norm	PCO2 ART	GEV, LogNorm
25 OH VIT D	GEV, Logistic			PH ART	Norm, GEV, Gamma, Logistic, LogNorm, Weibull
PH UA	GEV, Normal, Gamma, Logistic, LogNorm, Weibull			PH VEN	GEV, Norm, Gamma, Logistic, LorNorm, Weibull
ACR	GEV				

**Table 2:** Clinical evaluation of the PopKLD method for selecting cohorts. For three diseases, diabetes, chronic kidney disease and pancreatitis and three related laboratory measurements, glucose, creatinine and lipase, we compare the presence/absence of a disease identified by manual review with presence/absence of a disease identified using output from the PopKLD algorithm. We want to see positive correlation between a *low* KL-divergence and a *high* cluster purity because this implies that the model selected by the PopKLD method separated patients in a ways useful for identifying phenotypes and cohorts. Generally, the PopKLD method worked well identifying presence of a disease compared with other laboratory data based metrics. Most metrics worked well identifying absence of a disease compared with presence of a disease, a result that is expected because the low outlier error indicates absence whereas high outlier errors produce false positives.

Clinical evaluation of cluster purity of PopKLD selected cohorts			

Disease state	Model-defined cohort	KL-divergence	Purity (Proportion)
**Glucose collected in the AIM clinic**			
Diabetes	GEV() 10th decile, shape >0	**3.1** ⇐	$0.93(\frac{14}{15})$
Diabetes	GEV() 10th decile, shape <0	**3.1** ⇐	$0.93(\frac{14}{15})$
Diabetes	logn() 10th decile	4.3	$1(\frac{15}{15})⇐$
Diabetes	mean and standard deviation 10th decile	–	$0.53(\frac{8}{15})$
No Diabetes	GEV() 1st decile, shape >0	**3.1** ⇐	$0.93(\frac{14}{15})$
No Diabetes	GEV() 1st decile, shape <0	**3.1** ⇐	$0.93(\frac{14}{15})$
No Diabetes	logn() 1st decile	4.3	$1(\frac{15}{15})⇐$
No Diabetes	mean and standard deviation 1st decile	–	$1(\frac{15}{15})⇐$
**Creatinine collected in the AIM clinic**			
CKD	GEV() 10th decile, shape >0	**1.1** ⇐	$0.53(\frac{8}{15})$
CKD	GEV() 10th decile, shape <0	**1.1** ⇐	$0.8(\frac{12}{15})⇐$
CKD	logn() 10th decile	2.0	$0.53(\frac{8}{15})$
CKD	mean and standard deviation 10th decile	–	$0(\frac{0}{15})$
No CKD	GEV() 1st decile, shape >0	**1.1** ⇐	$1(\frac{15}{15})⇐$
No CKD	GEV() 1st decile, shape <0	**1.1** ⇐	$1(\frac{15}{15})⇐$
No CKD	logn() 1st decile	2.0	$1(\frac{15}{15})⇐$
No CKD	mean and standard deviation 1st decile	–	$1(\frac{15}{15})⇐$
**Lipase collected in the AIM clinic**			
Pancreatitis	GEV() 10th decile, shape	**73** ⇐	$0.27(\frac{4}{15})$
Pancreatitis	GEV() 10th decile, shape <0	**73** ⇐	$0.2(\frac{3}{15})$
Pancreatitis	logn() 10th decile	80	$0.87(\frac{13}{15})⇐$
Pancreatitis	mean and standard deviation 10th decile	–	$0(\frac{0}{15})$
no Pancreatitis	GEV() 1st decile, shape >0	**73** ⇐	$0.8(\frac{12}{15})$
no Pancreatitis	GEV() 1st decile, shape <0	**73** ⇐	$1(\frac{15}{15})⇐$
no Pancreatitis	logn() 1st decile	80	$1(\frac{15}{15})⇐$
no Pancreatitis	mean and standard deviation 1st decile	–	$0.73(\frac{11}{15})$

## Appendix A. Parameterized distributions

*Normal distribution*: is a member of the exponential class. The normal is important for at least two reasons. First, the normal distribution is universal because of the central limit theorem that states that summation or mean of any *iid* random variable with finite variance will have a normal distribution. The second reason why the normal family of distributions is important is that it is the *maximum entropy distribution* for a system whose mean and variance are known. If the normal distribution is the most representative model, then we can conclude that the process we are observing is: (i) generated by a normally distributed random variable (not likely because physiology generally does not have infinite support), (ii) the observed process is a sum of random variables (more likely because the original generating process can be nearly any random variable), or (iii) we can only resolve the mean and variance (as a consequence of maximum entropy).

*Log-normal distribution:* is the distribution of a random variable whose logarithm is normal distributed. Like the normal distribution, it is important for the same reasons in log coordinates. The log-normal distribution is the *multiplicative product* of positive *iid* random variables instead of the sum because it is in log coordinates (the log of a product is a sum). The log-normal distribution is the *maximum entropy probability distribution* for a system, *X*, whose mean and variance of *ln*(*X*) are known. The log-normal family of distributions is an intuitive choice for modeling EHR data because of its generative properties—the product of positive random variables—and because it is defined for only positive numbers.

*Generalized extreme value distributions.* (GEV) is a family of three distributions, GEV I, GEV II and GEV III, who are joined within a single equation. The GEV is the universal distribution of extrema of distributions according to the *extreme value theorem* and models properly normalized distributions of extrema of random variables if the extrema exist. The extreme value theorem, the extreme value analog of the central limit theory, shows that the GEV class of distributions is the *only* distribution family that models extrema of distributions. This universality makes the GEV a particularly important family, especially in the EHR context because it is often claimed that by measuring people only when why are sick, we are capturing the extrema of their physiology. It is important to note how the GEV and the Weibull, a subclass of the GEV, arrive at different parameter estimates, implying that the constraints limiting the GEV to the Weibull can have significant impact on the modeling estimates.

*Weibull distribution:* is one of the three types of extreme value distributions, GEV III and is a member of both the exponential and GEV families. Varying the parameters of the Weibull distribution interpolates between the exponential distribution and Rayleigh distribution while remaining within the exponential family. The variable in the Weibull distribution has been conceptualized as a distribution for particle size and more canonically in the extreme value context, a time to failure.

*Rayleigh distribution:* is a member of the exponential family, is defined for the positive real line, and is a special case of the Weibull which is therefore also a member of the GEV family. Moreover the Rayleigh distribution is a generalization of both the gamma or the chi distributions, depending on how the parameters are constrained. The interpretation of the Rayleigh distribution is as the magnitude, $\sqrt{x^{2}+y^{2}}$, of a two dimensional vector whose components are independent, normally distributed, and have equal variance. It is not easy to interpret glucose in this framework, but the Rayleigh distribution's relationship to other distributions helps to show how restricted parameterizations lead to different MLE-based estimates.

*Exponential distribution:* is a member of the exponential family of distributions. The exponential distribution represents a generative random process capturing the time between consecutive random events in Poisson process with no memory.

*Gamma distribution:* is a member of the exponential and generalized hyperbolic family of distributions. Generatively the gamma distribution models waiting times, such as times to death, of Poisson process. More importantly, the gamma distribution is the *maximum entropy distribution* for a process whose expected value is related to its univariate parameterization times a constant. Or, written differently, the gamma distribution is maximum entropy variable for processes whose mean-like and standard-deviation-like quantities are dependent [61].

*Logistic distribution:* is highly flexible and can be, depending on constraints on its parameters, a member of either or both the GEV or exponential families. The logistic distribution is notable because of its flexibility, because it is widely used in machine learning (e.g., in neural networks), because its cumulative probability density function of the logistic function, and because it is essentially a more flexible, normal distribution with fatter tails.

*Uniform distribution:* is the maximum entropy distribution when only a mean is known. Specifically, if we only have information about the mean and nothing else, the UD is the least biased distribution, and is essentially the distribution to beat. The uniform distribution is not a member of GEV or exponential family of distribution.

*Pareto:* is the family of distributions that follow a power-law and is not a member of either the exponential or the GEV family of distributions. Generally the Pareto distribution is used to model extrema with fat tail distributions. Power-law dependencies afford many interpretations and are common in nature. The most common interpretation of a power-law is the implication of scale independence because the functional dependence does not change over different scales of the independent variable. Like the log-linear distribution, it allows for the linearization of dependencies of variables that are only linear in log-log coordinates.

*t-distribution:* is an approximation of the normal distribution when the sample size is small and the variance is unknown. As the sample size is increased, the distribution tends toward a normal but generally has fatter tails than the normal distribution. The t-distribution can have a variable number of parameters or degrees of freedom, here we report results with three degrees of freedom but exampled it with between one and five. The t-distribution is a member of the much larger generalized hyperbolic distribution. The t-distribution is the *maximum entropy distribution* under the constraint that *E* [ln(*ν* + *X*^2^)] is constant, where *ν* is the number of degrees of freedom.
